# Capturing anatomy in computed tomography scans for genital pathology

**DOI:** 10.1007/s10140-024-02235-z

**Published:** 2024-05-31

**Authors:** Anna Chen, Allen Siapno, Tae-Hee Kim, Christopher Kanner, Tasha Posid, Taylor Goodstein

**Affiliations:** 1 The Ohio State University College of Medicine, Columbus, OH 43210 USA; 2 The Department of Urology, Ohio State University Wexner Medical Center, Columbus, OH 43210 USA; 3 The Department of Radiology, Ohio State University Wexner Medical Center, Columbus, OH 43210 USA

**Keywords:** Tomography, X-Ray computed, Pelvis, Patient safety, Diagnostic imaging, Genital pathology, Quality improvement, Surgery, Urology

## Abstract

**Purpose:**

In this cross-sectional study, we aimed to characterize how frequently the anatomy of interest (AOI) was excluded when evaluating genital pathology using the current CT pelvis protocol recommended by the American College of Radiology and evaluate how AOI exclusion affects patient management.

**Methods:**

We retrospectively reviewed medical records, using diagnosis and CPT codes, of patients admitted with genital pathology who obtained a CT scan at our institution from July 1, 2020–April 30, 2023. Baseline patient demographics were included. Data about each index CT scan (scan obtained at our institution) were recorded and assessed for exclusion of the AOI. Statistical analysis was performed to determine the rate of AOI exclusion and to compare patient management between patients with AOI excluded versus those without AOI exclusion.

**Results:**

113 presentations for genital pathology included an index CT scan and were included for analysis. Patients were primarily men (98%) with a mean age of 53.1 years (SD 13.9). The most common diagnoses were Fournier’s gangrene (35%), scrotal abscess (22%) and unspecified infection (19%). 26/113 scans (23%) did not capture the entire AOI. When the AOI was missed during the index scan, there was a higher rate of obtaining additional scans (38% vs. 21%), but a similar rate of intervention (77% vs. 63%) when compared to index scans that captured the entire AOI. 35 scans (31%) had protocol-extending instructions; index scans that captured the entire AOI were more likely to have specific protocol-extending instructions (38% vs. 8% p < 0.01).

**Conclusions:**

Creating a specific CT protocol for genital pathology could decrease the amount of inappropriate irradiation and improve AOI capture rates without relying on specific request for protocol deviation.

## Introduction

Genital infections have a high burden of disease and can lead to life-threatening complications [1]. Patients often present to the emergency department with pain, redness, swelling,  and tenderness of the genital region, which may be accompanied with fever and nausea and/or vomiting [2]. Fournier’s gangrene (FG) is a rare form of necrotizing soft tissue infection (NSTI) of the genital region that can be fatal if not quickly diagnosed and treated [3, 4]. Though clinical diagnosis is possible, FG may go unnoticed or misdiagnosed in its early stages because of initial superficial fascial and soft tissue involvement with little to no skin manifestations [5]. Physical exam findings of ecchymosis, crepitus, and necrotic tissue can indicate a more severe presentation [2]. Antibiotics and surgical debridement of FG to remove all affected tissue and prevent further rapid spread are the mainstay of management [2, 6]. Abscesses represent another infectious pathology that can develop in the genital region from unresolved bacterial infection and inflammation [7]. Other relevant genital pathologies include urinary and/or enteric fistulae and prosthetic device infection [8, 9]. 

Computed tomography (CT) has become a helpful adjunct for confirming genital pathology, identifying sources of infection, and evaluating disease extent to guide medical/surgical management [6, 10–17]. While simple radiography, ultrasound, and magnetic resonance imaging (MRI) may also be utilized in evaluation and diagnosis of genital pathology, CT imaging has been shown to be superior and is most commonly ordered due to its high specificity, high sensitivity, and short acquisition time [11]. These advantages are especially relevant in the diagnosis of urologic emergencies, such as FG. Key features of FG on CT imaging include fascial air and edema in the muscle, fascia, and/or subcutaneous layer in the genital region [7, 11]. 

The protocol for a CT of the pelvis used at our institution scans from the iliac crest through the ischial tuberosities. This is consistent with the parameters recommended by the American College of Radiology which states that “a CT of the pelvis extends from the iliac crest through just below the ischial tuberosities”, noting that “occasionally, more inferior extension of imaging may be required to fully image pelvic structures of concern” [18]. This protocol reduces radiation exposure to the testicles during routine examinations; however, it does not include a full view of the genital area, especially in men, which may limit utility and interpretability in the setting of genital pathology like FG [17]. We currently have no specific CT protocol for full inclusion of the genital area if genital-specific pathology is suspected, and full inclusion must be requested specifically (i.e. “extend scan to midthigh/knees”, “include full scrotum”, include genitals”) by either the ordering physician or the protocoling radiologist. The lack of this specific protocol may be a potential source of error for the early detection of genital pathology, which is especially relevant for a critical disease such as FG.

This multidisciplinary cross-sectional study was conducted to characterize how frequently the genital anatomy of interest (AOI) was missed using the current CT pelvis protocol. Secondarily, we sought to identify how often specific requests are made by either the ordering physician or radiologist, and if this improves rates of AOI inclusion. Finally, we wanted to evaluate the effect of missing anatomy on procedure timing or rates of repeat imaging. We hypothesized that the rate of AOI exclusion is less when specifying language is utilized in the CT protocol by the ordering physician or protocoling radiologist. The purpose of this study was to provide evidence for creating a genital pathology–specific CT protocol that scans well below the ischial tuberosities.

## Methods

This descriptive cross-sectional study followed the guidelines set by Strengthening the Reporting of Observational Studies in Epidemiology (STROBE) [19]. 

### Study population

Following IRB approval, diagnosis codes for various genital pathologies including FG, abscess, inflammatory conditions of the scrotum/perineum and inflammatory conditions involving prosthetic devices in the genital tract, as well as the CPT code for genital debridement were used to identify patients presenting to our institution from July 1, 2020 – April 30, 2023. Inclusion criteria included patients aged 18 years and older who had genital pathology as above (e.g. FG, abscess, fistula) and obtained a CT scan at our institution during the same presentation (index CT scan). Patients were excluded if they did not receive at least one CT scan at our institution during their presentation for genital pathology.

### Measures of interest

Baseline demographics, clinical characteristics at presentation, and index CT scan characteristics were collected on all patients. The primary measure of interest was investigator-determined AOI and whether it was included in its entirety on the index CT scan. The AOI was determined based on the assessing physician’s history of presenting illness and confirmed by the reason for exam listed in the CT imaging order prior to viewing the index scan. For CT scans where it was unclear whether the AOI was captured fully, a second investigator reviewed the imaging independently to prevent potential confirmation bias. Secondary measures included the documented ordering reason for the index CT scan, whether the ordering provider or the radiologist added directive language (e.g., “scan to midthigh” or “include full scrotum”) in the ordering comments or the protocoling instructions, and whether inclusion of specifying language in the CT protocol improved capture of AOI. Tertiary measures included whether multiple scans were obtained (and if so, how many), and whether the patient underwent any surgical or procedural interventions during the admission. Additional measures of interest included whether the patient was transferred from an outside facility (OSH) and whether they received any CT scans from the outside facility before being transferred to an affiliated facility. For patients who obtained multiple scans, only the initial CT scan obtained was used for analysis of tertiary measures. Data collected was stored and managed on an electronic REDCap database provided by our institution [20, 21]. 

### Statistical analysis

Data analysis was performed using SPSS Statistics software (IBM SPSS Statistics for Windows, version 29.0. Armonk, NY: IBM Corp). Data are presented as means (standard deviations) or proportions (percentages). Demographics and clinical characteristics of the study population was analyzed via descriptive and frequency statistics. Per study objectives, analyses were performed comparing group differences via Fisher’s exact test or Chi-square test for categorical variables or independent t-tests for continuous variables. Significance was defined as *p* < 0.05. Patients with missing data were included in the analyses; missing data points were identified as “No Data” in the resulting tables.

## Results

**Fig. 1:**
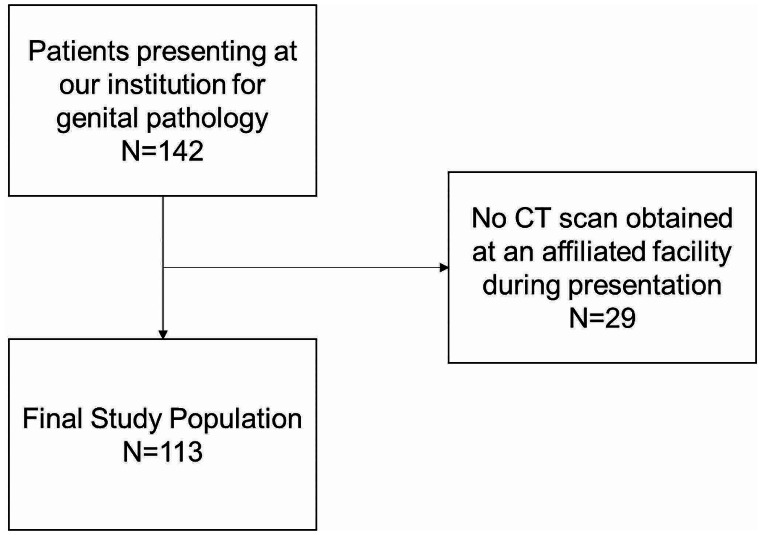
Study population flowchart with exclusion criteria

During the study period, there were 142 presentations at our institution for genital pathology. 113 of these instances included an index CT scan and were included for analysis (Fig. 1). Patients who did not receive a CT scan at our institution were excluded from the study population. Most study subjects were male (98%) and obese (average BMI 34.1 [SD 10.1]), with a mean age of 53.1 years. Fournier’s gangrene was the most common presenting diagnosis (35%), followed by scrotal abscess (22%), and unspecified infection (19%). Out of 30 transfers to our institution, 22 (73%) had already received a CT scan at the outside hospital. Demographics and clinical information on the patient cohort are included in Table 1.

**Table 1:** Patient demographics and clinical characteristics (*N* = 113)

		AOI excluded *N* = 26 (%)	AOI included *N* = 85 (%)	*P* value
Age, Mean (SD)		56.8 (11.6)	52.1 (14.4)	
Sex				0.43
	Male	26 (100)	85 (100)	
	Female	0 (0)	2 (2)	
BMI, Mean (SD)		33.1 (8.6)	34.4 (10.5)	0.94
	Underweight (BMI < 18.5)	0 (0)	1 (1)	
	Normal weight (18.5–24.9)	4 (15)	15 (17)	
	Overweight (25-29.9)	6 (23)	19 (22)	
	Obese (30-34.9)	7 (27)	18 (21)	
	Morbidly obese (> 35)	9 (35)	34 (39)	
Diagnosis				0.91
	Fournier’s Gangrene/NSTI	9 (35)	31 (35)	
	Scrotal Abscess	5 (19)	20 (23)	
	Perineal Abscess	3 (12)	5 (6)	
	Vulvar abscess	0 (0)	1(1)	
	Fistula	1 (4)	3 (3)	
	Infection, otherwise not specified	4 (15)	18 (21)	
	Other*	4 (15)	9 (10)	
OSH Transfer				0.51
	Yes	9 (35)	21 (24)	
	No	17 (65)	65 (75)	
	No data	0	1 (1)	
CT Scan at OSH (*n* = 30)				0.72
	Yes	7 (78)	15 (71)	
	No	2 (22)	6 (29)	

*Other included buried penis, hydrocele, and scrotal cyst

Of the 113 index CT scans, 68 (60%) were CT scans of the abdomen and pelvis and 45 (40%) were CT scans of the pelvis only. The scrotum was identified as the AOI by the investigator in 93 scans (82%) and the perineum was the AOI in 44 scans (39%). Overall, 26 (23%) scans did not capture the AOI; the scrotum was the AOI in 25 of these scans. There were no statistically significant differences in demographic information and clinical characteristics between those scans that included versus excluded the AOI (Table 1). FG/NSTI was the most common ordering reason (39%), followed by scrotal abscess (16%). Out of the 44 scans ordered for FG, 25 (57%) resulted in a concordant diagnosis. Scans ordered for scrotal abscess confirmed this diagnosis in 8 out of 18 scans (44%). Details on index CT scans are included in Table 2.

**Table 2:** Index CT scan characteristics (*N* = 113)

		*N* (%)
Surgical team consulted before CT	Yes	40 (35)
	No	66 (58)
	No data	7 (6)
Type of scan		
	CT Pelvis	45 (40)
	CT Abdomen/Pelvis	68 (60)
Reasons for exam		
	Fournier’s gangrene/NSTI	44 (39)
	Scrotal abscess	18 (16)
	Perineal abscess	8 (7)
	Penile abscess	2 (2)
	Groin abscess	3 (3)
	Groin infection	2 (2)
	Genital infection	3 (3)
	Other*	65 (58)
Reason for scan concordance with diagnosis		
	Fournier’s gangrene/NSTI (*n* = 44)	25 (57)
	Scrotal abscess (*n* = 18)	8 (44)
	Perineal abscess (*n* = 8)	1 (13)
Specific instructions provided by ordering provider		
	Yes	26 (23)
	No	87 (77)
Specific instructions provided by radiologist		
	Yes	26 (23)
	No	87 (77)
Indicated AOI		
	Scrotum	93 (82)
	Penis	23 (20)
	Perineum	44 (39)
	Vulva	1 (1)
Entire AOI included on CT		
	Yes	87 (77)
	No	26 (23)
AOI excluded on CT		
	Scrotum	25
	Penis	4
	Perineum	6
	Vulva	0

*Other reasons for scans included lower abdominal pain, scrotal pain, hernia, soft tissue swelling, scrotal erythema, fever, sepsis

A total of 35 scans (31%) had protocol-extending instructions. Specific protocol-extending requests by the ordering provider were provided in 26 (23%) scans. In 17 of these 26 scans (65%), the protocoling radiologist also provided protocol-extending instructions; the AOI was missed in one of the 9 scans for which the radiologist did not provide the same protocol-extending instructions as the provider. There were 9 scans (10%) where the radiologist provided protocol-extending instructions without a protocol-extending request from the ordering provider; the AOI was missed in zero of these scans. We found that index scans that captured the entire AOI were more likely to have specific protocol-extending instructions by the ordering physician or radiologist (38% vs. 8% *p* < 0.01). Specifically, instructions such as “scan to mid-thigh/knees” (23% vs. 4%, *p* = 0.04) and “include scrotum” (21% vs. 4%, *p* = 0.07) were more often provided in scans that captured the AOI. When the AOI was not captured on the initial scan, there was a higher rate of obtaining additional scans during the patient’s admission (38% vs. 21%), and this difference neared significance (*p* = 0.08). The rate of intervention was similar between scans with incomplete vs. complete capture of AOI (77% vs. 63%, *p* = 0.24). Of the 75 procedures performed, 12 (16%) were performed at the bedside and 63 (84%) were in the OR. We also found that while there were no significant differences between AOI inclusion and exclusion when specific reasons for scans were selected, there was a statistically significant increase in AOI exclusion when the reason for scan was “other,” which included a myriad of vague clinical situations (Table 2; *p* = 0.006).

The surgical team was consulted prior to CT completion for 40 (35%) presentations (Table 2). The timing of consult to the surgical team (whether consult was placed before or after scan was obtained) did not affect rates of inclusion of protocol-extending instructions. Analyses on the differences in instructions, repeat scans, and interventions are included in Table 3.

**Table 3:** Differences in instructions,repeat scans and interventions by AOI exclusion

		AOI exclusion (*N* = 26)	Complete AOI inclusion (*N* = 87)	
		N (%)	N (%)	*P* value
Reason for scan^*				
	Fournier’s gangrene/NSTI	8 (31)	36 (41)	0.37
	Scrotal abscess	2 (8)	16 (18)	0.24
	Perineal abscess	0 (0)	8 (9)	0.11
	Penile abscess	1 (4)	1 (1)	0.41
	Groin abscess	1 (4)	2 (2)	0.55
	Groin infection	0 (0)	2 (2)	1.0
	Genital infection	0 (0)	3 (3)	1.0
	Other	21 (80)	44 (50)	0.006
Instructions provided by orderingpPhysician or radiologist *				
	Yes	2 (8)	33 (38)	< 0.01
	No	24 (92)	54 (62)	
Instructions *				
	“Scan to mid-thigh/knees”	1 (4)	20 (23)	0.04
	“Include scrotum”	1 (4)	18 (21)	0.07
	“Include genitals”	0 (0)	3 (3)	1.0
Repeat scans done *				
	Yes	10 (38)	18 (21)	0.08
	No	16 (62)	69 (79)	
Procedure done *				
	Yes	20 (77)	55 (63)	0.24
	No	6 (23)	32 (37)	
Procedure type (*n* = 75)				
	Bedside procedure	5 (25)	7 (13)	
	Operative intervention	15 (75)	48 (87)	

^Multiple reasons for scan can be selected

* Fisher’s Exact Test

## Discussion

In this study, we sought to determine the rates of missed anatomy on standard CT scans obtained for genital pathology and found that this happens in about a quarter of scans obtained for this indication. As far as the authors are aware, this is the first study examining genital capture of CT scans in the literature. This research establishes the foundation for developing a genital pathology–specific CT protocol at our institution.

There were several interesting findings from this study. We found that a CT abdomen/pelvis was more commonly ordered over a CT pelvis (60% vs. 40%), which, for the purposes of assessing genital pathology, unnecessarily irradiates the abdominal organs. Eliminating unnecessary radiation exposure is a key concept in the development of CT protocols, and the reason why a CT pelvis does not extend to universally include the testicles [22–24]. Developing a genital pathology–specific CT protocol would not include the abdomen, thus eliminating unnecessary radiation exposure of the abdominal organs. Since patients with missed anatomy were more likely to obtain multiple scans during their admission (though not significant), a genital pathology–specific CT protocol could potentially decrease the rates of missed anatomy and also potentially reduce the radiation exposure from additional scans.

Though not the primary quality improvement focus of this study, we also found that 19% of presenting patients already had a scan from an outside hospital prior to arrival at our institution. Whether or not images from these scans were available or if they included full AOI was not measured in this study, but this represents a potential area for improvement that could also decrease unnecessary radiation exposure.

Unsurprisingly, inclusion of protocol-extending language decreased the rates of missed AOI, but only 38% of scans included any protocol-extending instruction. It was notable that protocol-extending request made by the ordering provider did not always lead to a protocol-extending instruction from the radiologist, though this did not seem to drastically affect missing AOI (only 1 of 9 scans fitting these criteria had missing AOI), suggesting that the CT technologist independently extends the protocol when requested by the ordering provider. Additionally, the protocoling radiologist requested a protocol extension without a request from the ordering provider only 10% of the time, which excludes radiologist discretion as a reliable method of extending scans when indicated based on reason for ordering.

While there is a high rate of concordance between reason for scan and diagnosis specifically for FG/NSTI and scrotal abscess, they only represent a small fraction of the entire study. It is also important to note the high rate of discordance between the reason for ordering scan and ultimate diagnosis, which further highlights the need for comprehensive imaging when genital pathology is suspected. There were 79 (70%) instances where the reasons for ordering the scan did not match the final diagnosis. The 65 (58%) cases where the reason for scan was designated “other”, included general and non-specific descriptors in the reason for scan (e.g. “lower abdominal pain”, “infection”, “skin lesion”, “fever”, “sepsis”). For the 26 index scans where AOI was excluded, 21 had a reason of scan investigator-designated as “other”. As a result, utilizing vague descriptors in the reason for exam when ordering CT imaging could lead to increased rates of AOI exclusion. The current mode of ordering CT imaging heavily relies on the proactivity of the ordering physician and radiologist; however, developing a comprehensive imaging protocol specifically for genital pathology may help eliminate these concerns and improve acquisition of AOI.

We are limited by the retrospective nature of this study and the relatively small sample size of CT scans. Additionally, 98% of patients in this study were male, limiting the applicability of this data to female patients. It is thus unclear if a genital-specific CT protocol would be of any benefit in female patients, though it is unlikely to lead to clinically significant irradiative consequences since the female gonads are universally irradiated from a CT pelvis. Some argument could be made for limiting the proximal extent of a genital-specific CT protocol to avoid scanning the pelvic organs completely, but this study was not designed to determine the feasibility of this, as we did not assess the likelihood of missing significant pathology in the setting of a proximally limited scan.

Furthermore, while this study examined the utilization of standard CT pelvis protocols on several diagnosed urologic conditions, the only CPT code utilized to identify these patients was for genital debridement. This could mean that we neglected to capture patients who underwent other operative interventions such as simple I&D, device explantation, or diversion for fistulae. However, utilizing the diagnostic codes for these conditions was felt to be more accurate for capturing these patients, as many are managed non-operatively initially or with bedside procedures. Though this study focused specifically on operative management for FG, creation of a genital-specific CT protocol would almost certainly have broad applications to all genital pathology.

This study was not designed to evaluate ultrasound evaluation of these pathologies and how diagnoses made by ultrasound might relate to CT scan results. Many patients who present to the ED with these types of conditions are initially (and sometimes only) evaluated by ultrasound, but the influence of ultrasound on CT ordering or patient management was not measured in the current study.

Since the completion of this cross-sectional study, we are working to implement a genital pathology–specific CT protocol at our institution, which would include a standard CT pelvis that starts from the iliac crest and extends to mid-thigh. Future directions of this work will include assessing rates of missed AOI after developing a genital–pathology-specific CT protocol at our institution. Ultimately, we hypothesize that development of a genital pathology–specific CT protocol will improve inclusion rates of AOI when genital pathology is suspected, reduce radiation exposure from additional CT imaging, improve surgical planning and medical management, and contribute to high-value patient care.

## References

[1] McArthur M, Patel M (2023) A pictorial review of genitourinary infections and inflammations. Clin Imaging 104:110013. 10.1016/j.clinimag.2023.110013[published Online First: 20231026] 37918136 10.1016/j.clinimag.2023.110013

[2] Davis JE, Silverman M (2011) Scrotal emergencies. Emerg Med Clin North Am 29(3):469–484. 10.1016/j.emc.2011.04.011 21782069 10.1016/j.emc.2011.04.011

[3] Smith GL, Bunker CB, Dinneen MD (1998) Fournier’s gangrene. Br J Urol 81(3):347–355. 10.1046/j.1464-410x.1998.00532.x 9523650 10.1046/j.1464-410x.1998.00532.x

[4] Hong KS, Yi HJ, Lee RA et al (2017) Prognostic factors and treatment outcomes for patients with Fournier’s gangrene: a retrospective study. Int Wound J 14(6):1352–1358. 10.1111/iwj.12812[published Online First: 20170925] 28944569 10.1111/iwj.12812 PMC7950045

[5] Short B (2018) Fournier gangrene: an historical reappraisal. Intern Med J 48(9):1157–1160. 10.1111/imj.14031 30182399 10.1111/imj.14031

[6] Chennamsetty A, Khourdaji I, Burks F et al (2015) Contemporary diagnosis and management of Fournier’s gangrene. Ther Adv Urol 7(4):203–215. 10.1177/1756287215584740 26445600 10.1177/1756287215584740 PMC4580094

[7] Gossner J (2024) A pictorial review of scrotal and penile pathology on computed tomography. Emerg Radiol. 10.1007/s10140-023-02198-7[published Online First: 20240109] 38194213 10.1007/s10140-023-02198-7

[8] Shyam DC, Rapsang AG (2013) Fournier’s gangrene. Surgeon 11(4):222–232. 10.1016/j.surge.2013.02.001[published Online First: 20130408] 23578806 10.1016/j.surge.2013.02.001

[9] Yu M, Robinson K, Siegel C et al (2017) Complicated genitourinary tract infections and mimics. Curr Probl Diagn Radiol 46(1):74–83. 10.1067/j.cpradiol.2016.02.004[published Online First: 20160211] 26995297 10.1067/j.cpradiol.2016.02.004

[10] Rajan DK, Scharer KA (1998) Radiology of Fournier’s gangrene. AJR Am J Roentgenol 170(1):163–168. 10.2214/ajr.170.1.9423625 9423625 10.2214/ajr.170.1.9423625

[11] Ballard DH, Mazaheri P, Raptis CA et al (2020) Fournier Gangrene in men and women: appearance on CT, Ultrasound, and MRI and what the Surgeon wants to know. Can Assoc Radiol J 71(1):30–39. 10.1177/0846537119888396[published Online First: 20200128] 32063012 10.1177/0846537119888396 PMC7047600

[12] Ballard DH, Raptis CA, Guerra J et al (2018) Preoperative CT findings and Interobserver Reliability of Fournier Gangrene. AJR Am J Roentgenol 211(5):1051–1057. 10.2214/AJR.18.19683[published Online First: 20180807] 30085837 10.2214/AJR.18.19683 PMC6451931

[13] Leichtle SW, Tung L, Khan M et al (2016) The role of radiologic evaluation in necrotizing soft tissue infections. J Trauma Acute Care Surg 81(5):921–924. 10.1097/TA.0000000000001244 27602893 10.1097/TA.0000000000001244

[14] Levenson RB, Singh AK, Novelline RA (2008) Fournier gangrene: role of imaging. Radiographics 28(2):519–528. 10.1148/rg.282075048 18349455 10.1148/rg.282075048

[15] Sherman J, Solliday M, Paraiso E et al (1998) Early CT findings of fournier’s gangrene in a healthy male. Clin Imaging ; 22(6) 10.1016/s0899-7071(98)00073-4 9876913

[16] Tonolini M, Ippolito S (2016) Cross-sectional imaging of complicated urinary infections affecting the lower tract and male genital organs. Insights Imaging 7(5):689–711. 10.1007/s13244-016-0503-8[published Online First: 20160607] 27271509 10.1007/s13244-016-0503-8 PMC5028337

[17] Wongwaisayawan S, Krishna S, Haroon M et al (2020) Fournier gangrene: pictorial review. Abdom Radiol (NY) 45(11):3838–3848. 10.1007/s00261-020-02549-9 32342151 10.1007/s00261-020-02549-9

[18] Brooks OR, Kurian J, Megibow A et al ACR-SABI-SAR-SPR practice parameter for the performance of computed tomography (CT) of the Abdomen and computed tomography (CT) of the Pelvis. 2021(46)

[19] von Elm E, Altman DG, Egger M et al (2014) The strengthening the reporting of Observational studies in Epidemiology (STROBE) Statement: guidelines for reporting observational studies. Int J Surg 12(12):1495–1499. 10.1016/j.ijsu.2014.07.013[published Online First: 20140718] 25046131 10.1016/j.ijsu.2014.07.013

[20] Harris PA, Taylor R, Thielke R et al (2009) Research electronic data capture (REDCap)--a metadata-driven methodology and workflow process for providing translational research informatics support. J Biomed Inf 42(2):377–381. 10.1016/j.jbi.2008.08.010[published Online First: 20080930] 10.1016/j.jbi.2008.08.010 PMC2700030 18929686

[21] Harris PA, Taylor R, Minor BL et al (2019) The REDCap consortium: building an international community of software platform partners. J Biomed Inf 95:103208. 10.1016/j.jbi.2019.103208[published Online First: 20190509] 10.1016/j.jbi.2019.103208 PMC7254481 31078660

[22] Tarin TV, Sonn G, Shinghal R (2009) Estimating the risk of cancer associated with imaging related radiation during surveillance for stage I testicular cancer using computerized tomography. *J Urol* ;181(2):627 – 32; discussion 32 – 3. 10.1016/j.juro.2008.10.005 [published Online First: 20081216] 10.1016/j.juro.2008.10.005 19091344

[23] Brenner DJ, Hall EJ (2007) Computed tomography–an increasing source of radiation exposure. N Engl J Med 357(22):2277–2284. 10.1056/NEJMra072149 18046031 10.1056/NEJMra072149

[24] Neisius A, Wang AJ, Wang C et al (2013) Radiation exposure in urology: a genitourinary catalogue for diagnostic imaging. J Urol 190(6):2117–2123. 10.1016/j.juro.2013.06.013[published Online First: 20130611] 23764073 10.1016/j.juro.2013.06.013

